# Psoas compartment block efficacy and safety for perioperative analgesia in the elderly with proximal femur fractures: a randomized controlled study

**DOI:** 10.1186/s12871-021-01473-9

**Published:** 2021-10-25

**Authors:** Kateryna Bielka, Iurii Kuchyn, Igor Tokar, Valerii Artemenko, Uliana Kashchii

**Affiliations:** 1 Postgraduate Department of Surgery, Anesthesiology and Intensive Care, Bogomolets National Medical University, 13 T. Shevchenko Boulevard, Kiev, 01601 Ukraine; 2 Department of Anesthesiology and Intensive Care, Medical Center “Into-Sana”, Varnenska street 2, Odesa, 65065 Ukraine

**Keywords:** Postoperative pain, Psoas compartment block, Proximal femur fracture

## Abstract

**Background:**

Proximal femur fractures are most common fractures in the elderly and associated with significant mortality and morbidity, with high economic and social impact. Perioperative pain management influence outcomes and mortality after surgery with early mobilization being possible. The goal of the study was to compare the efficacy and safety of the psoas compartment block (PCB) with spinal and general anesthesia.

**Methods:**

We included 90 patients in this randomized controlled study and divided them into three groups. For patients in group 1 ultrasound-guided PCB with bupivacaine 0.125% 6–8 ml / h was performed. Intraoperative anesthesia was provided with PCB and a sciatic nerve block. Postoperative analgesia include prolonged CPB with bupivacaine 0.125% 6–8 ml / h. In group 2 intraoperative spinal anaesthesia were performed. Group 3 patients underwent general sevoflurane inhalation anaesthesia with fentanyl infusion for analgesia. All patients received paracetamol 3 g/day and dexketoprofen 75 mg/day during hospitalization. On-demand, nalbuphine 5 mg SC was used for analgesia. Efficacy outcomes were the ICU length of stay and the total duration of hospitalization, number of patients who had severe pain after surgery, incidence of on-demand analgesia, sleep quality, postoperative mobilization time. Safety outcomes include complication incidence.

**Results:**

There were no differences in the duration of ICU stay - gr.1 72 [70–75], gr.2 74 [72–76], gr.3 72 [70–75] hours respectively (*p* = 0.29), and the total duration of hospitalization - gr.1144 [170–184], gr.2170 [148–188], gr.3178 [144–200] hours respectively. Patients in gr.1 had significantly lower nalbuphine consumption in the first 24 h after surgery and total during hospitalization (0 [0–5] mg versus 15 [10–20] and 20 [15–25] mg in the first 24 h in groups 2 and 3, respectively (*p* < 0.001). Gr. 1 had lower number of patients with severe pain (10% vs. 47 and 60% in groups 2 and 3, respectively, *p* < 0.05), lower number of on demand analgesia (0 [0–1] vs. 3 [2–4] and 4 [3, 4] in groups 2 and 3, respectively), better sleep quality (8 [7–9] vs. 6 [5–7] and 4 [3, 4] in groups 2 and 3, respectively, *p* < 0.001), significantly faster mobilization after surgery – sitting in bed and getting to his feet. MINS was diagnosed significantly more often in gr. 2 and 3 compared with gr. 1 (OR 9 95 CI 1,01–77, *p* = 0,048 for gr. 2 and OR 11 95 CI 1,2–91, *p* = 0, 03 for gr. 3). However, none of the patients had symptoms of myocardial ischemia and was not diagnosed with myocardial infarction. There were no difference in the incidence of nosocomial pneumonia and delirium.

**Conclusion:**

Perioperative PCB in elderly patients with a proximal femur fracture could be an effective analgesia technique, as it decrease the number of patients with severe pain, need for on demand analgesia and opioid consumption. PCB also decrease the incidence of opioid-associated nausea and vomiting, comparing to general anesthesia, and increase the number of patients, who was mobilized in the 1st day (sitting) and 2nd day (getting up) after surgery. PCB may reduce the incidence of MINS, although to assess this outcome more studies are needed.

**Trial registration:**

Clinicaltrials.gov: NCT04648332, first registration date 1/12/2020.

**Supplementary Information:**

The online version contains supplementary material available at 10.1186/s12871-021-01473-9.

## Background

There are approximately 1 million femur fractures in the world each year [1] with significant impact on life expectancy and quality [2, 3], with high risk of respiratory, cardiac and thrombotic complications. Postoperative mortality may vary between 7 and 11% at 1 month, 16 and 28% at 6 month and 22 and 37% at 1 year [4]. Surgical treatment, perioperative care and early mobilization are important and associated with mortality, incidence of complications, length of hospital stay and survival [5].

Every third patient with femur fracture has severe pain, and another 30% moderate pain [6]. While effective perioperative pain management is associated with significantly better outcomes: reduced duration of hospitalization and the risk of delirium, early mobilization, lower risk of respiratory and cardiac complications [6].

The most common techniques of perioperative analgesia for proximal femur fractures are systemic analgesia, neuraxial (epidural) analgesia and peripheral nerve blocks - psoas compartment block. Intraoperatively, respectively, anaesthesia is provided by general anaesthesia, neuraxial (spinal) anaesthesia or compartment psoas block in combination with a sciatic nerve block. Systemic analgesia is often limited in this group of patients due contraindications (like chronic renal disease for nonsteroidal anti-inflammatory drugs (NSAIDs) or the development of side effects (respiratory depression, nausea and vomiting, sedation). Neuraxial anaesthesia also had limitations due to risk of hemodynamic complications (hypotension, bradycardia), which may lead to postoperative myocardial and renal injury, and contraindications in patients who already receive anticoagulant or antiplatelet therapy.

Psoas compartment block is a peripheral regional technique of anaesthesia and analgesia, which provides a block of the main components of the lumbar plexus - the femoral, lateral cutaneous nerve of the femur and sciatic nerve. In combination with the sciatic nerve block, the psoas compartment block provides effective anaesthesia of the entire lower extremity, with better hemodynamic stability, compared to epidural anaesthesia [7, 8].

The aim of our study was to compare the effectiveness and safety of different techniques of perioperative anaesthesia and anaesthesia in patients with fractures of the proximal femur: general anaesthesia with systemic postoperative analgesia, neuraxial (spinal) anaesthesia with systemic postoperative analgesia and prolonged compartment psoas block in combination with a sciatic nerve block (intraoperatively).

## Materials and methods

**Fig. 1:**
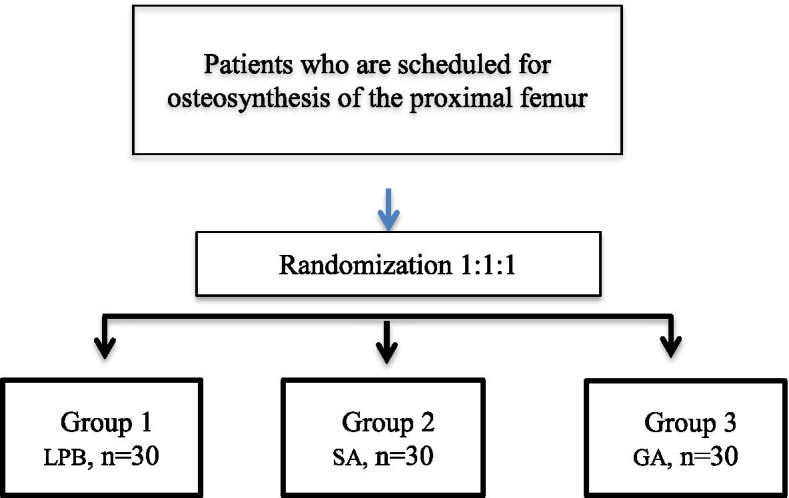
Distribution of patients in study groups: PCB - psoas compartment block, SA - spinal anesthesia, GA - general anesthesia

A randomized controlled trial was conducted from January 2018 to August 2019 at the Into-Sana Medical Center (Odessa, Ukraine). The study design was approved by the Ethical Committee at Bogomolets National Medical University. Patients who planned osteosynthesis of the proximal femur and who met the inclusion criteria were randomized to 3 study groups in a 1:1 ratio (Fig. 1) using random assignment in blocks of four. The randomization sequence was generated using a computer algorithm [9]. Randomization and data analysis were conducted by an independent blinded member of the research team.

Criteria for inclusion in the study were: signed informed consent, age over 18 years. Exclusion criteria were: patient age less than 18 years, patient refusal, pregnancy and lactation, history of opiate addiction, severe comorbidities (traumatic brain injury; acute stroke; dementia; acute cerebrovascular accident; chronic heart failure (New York Heart Association Functional Classification, NYHA, class III-IV), respiratory failure, renal failure with decreased creatinine clearance less than 30 ml / min / 1.73 m2, hepatic insufficiency class C according to Child-Pugh).

For patients in group 1, after including in the study, ultrasound-guided Shamrock CPB with bupivacaine 0.125% 6–8 ml / h was performed. Intraoperative anesthesia was provided with a bupivacaine bolus of 0.5% 200 mg in a lumbar catheter and a sciatic nerve block (neurostimulator identification) with 1.5% 450 mg of lidocaine. Postoperative analgesia include prolonged CPB with bupivacaine 0.125% 6–8 ml / h. Also paracetamol 3 g/day and dexketoprofen 75 mg/day was prescribed.

For patients in group 2 and 3 preoperative analgesia include paracetamol 3 g/day and dexketoprofen 75 mg/day. On-demand, nalbuphine 5 mg SC was used for analgesia.

In group 2 intraoperative spinal anaesthesia were performed at the level of L3-L4 with hyperbaric bupivacaine 10–15 mg. Patients in groups 1 and 2 receive intraoperative sedation with propofol 1% with a target level of sedation RASS from 0 to − 2.

Group 3 patients underwent general sevoflurane inhalation anaesthesia with fentanyl infusion for analgesia.

All patients received paracetamol 3 g/day and dexketoprofen 75 mg/day during hospitalization. On-demand, nalbuphine 5 mg SC was used for analgesia.

The primary efficacy outcome was: postoperative nalbuphine consumption during first 24 h and cumulative during hospital stay. SC injection of 5 mg nalbuphine was used as analgesic on-demand.

The secondary efficacy outcomes were:

- ICU length of stay and the total duration of hospitalization
- number of patients who had severe pain after surgery
- incidence of on-demand analgesia (nalbuphine 5 mg SC)
- sleep quality (from 0 to 10, where 0 - very bad / no sleep, 10 - excellent sleep)
- postoperative mobilization time (sitting in bed and getting to his feet)

The safety assessment criteria were complications and side effects throughout the perioperative period. Criteria for myocardial injury after noncardiac surgery (MINS) were increased highly sensitive troponin T (hsTnT) more than 20 ng /l with or without symptoms of ischemia. We did not include patients when increased troponin was thought to be associated with other noncardiac causes (sepsis, pulmonary embolism) or a consequence of chronic myocardial injury (hsTnT before operation 20 ng /l and more) [10]. HsTnT was performed for all patients before and in 48 h after surgery. Criteria for nosocomial pneumonia were new pulmonary infiltrates of infectious origin (with fever, sputum, leucocytosis, procalcitonin elevation and decreased oxygenation) that occurred 48 h or later after hospitalization [11]. The criteria of delirium were considered: disturbance of attention and consciousness; changes that have developed acutely (hours or days) and fluctuate during the day; cognitive impairment (memory, speech, orientation, perception, vision); there is no evidence that this condition has developed as a result of a medical condition, intoxication or withdrawal of certain substances, side effects of drugs [12].

All patients underwent intraoperative monitoring: ECG monitoring, blood pressure and pulse oximetry, BIS (group 3), capnography (group 3), postoperatively performed round-the-clock monitoring of vital signs. Pain was measured every 2 h during first 48 h after surgery (excluding night time) with numeric pain rating scale (NPRS), where 0 – no pain, 10 – most severe pain you can imagine. The criteria for severe postoperative pain was pain measured as 7 or more during 30% or more time after surgery.

Sample size was calculated using MedCalc Software version 16.8.4 (MedCalc Software bvba, Acacialaan 22, 8400 Ostend, Belgium). Based on minimum mean difference of 25% in morphine consumption [13] with α = 0.01 and β = 0.20, sample size for each group was estimated as 20. So, we include 30 patients in each group. Statistical analysis was performed with Statistica 8.0 programs. Categorical data are presented as proportions, continuous - as the median and 25–75 quadrantiles. The Chi-square test was used to determine the normality of the data distribution in the sample, and most of the results in the study are nonparametric. The Kruskal-Wallis test to compare differences between multiple groups, Mann-Whitney test to compare differences between two groups and the Fisher double test to compare proportions were used. The probability of error (p) was considered insignificant at *p* < 0.05.

## Results

The study included 90 patients (30 in each group, respectively). Patients in different groups did not differ statistically in demographics and comorbidities (Table 1). There were also no differences in the duration of hospitalization in the intensive care unit - gr.1 72 [70–75], gr.2 74 [72–76], gr.3 72 [70–75] hours respectively (*p* = 0.29, Kruskal-Wallis test), and the total duration of hospitalization - gr.1144 [170–184], gr.2170 [148–188], gr.3178 [144–200] hours respectively (*p* = 0.5, Kruskal-Wallis test).

**Table 1:** Demographic characteristics of patients in study groups

Indicator/group	Group 1	Group 2	Group 3	*p*
Gender, female, n (%)	21/9(70)	21/9 (70)	22/8 (73)	*p* > 0,05^1^
Age*	72 [68–73]	72 [70–73]	73 [72–74]	*p* = 0,14^2^
Concomitant pathology:				
Diabetes mellitus, n (%)	3/27 (10)	4/26 (13)	4/26 (13)	*p* > 0,05^1^
Hypertension, n (%)	6/24 (20)	7/23 (23)	9/21 (30)	*p* > 0,05^1^
Chronic kidney disease, n (%)	2/28 (7)	2/28 (7)	2/28 (7)	*p* > 0,05^1^
COPD, n (%)	2/28 (7)	1/29 (3)	2/28 (7)	*p* > 0,05^1^
Other:	6/24 (20)	8/22 (27)	6/24 (20)	*p* > 0,05^1^

Unless specified otherwise, values are expressed as medians, with 25–75% interquartile ranges in parentheses. *COPD* Chronic obstructive pulmonary disease

^1^ - Fisher’s exact test, ^2^ - Kruskal-Wallis test

Patients in group 1 had significantly lower nalbuphine consumption in the first 24 h after surgery and total during hospitalization (0 [0–5] mg versus 15 [10–20] and 20 [15–25] mg in the first 24 h in groups 2 and 3, respectively (*p* < 0.001), the lowest number of patients with severe pain (10% vs. 47 and 60% in groups 2 and 3, respectively, *p* < 0.05), lower frequency of on-demand analgesia (0 [0–1] vs. 3 [2–4] and 4 [3, 4] in groups 2 and 3, respectively), better sleep quality (8 [7–9] vs. 6 [5–7] and 4 [3, 4] in groups 2 and 3, respectively, *p* < 0.001), significantly faster mobilization after surgery – sitting in bed and getting to his feet (see Table 2).

**Table 2:** Efficacy outcomes

Indicator/group	Group 1	Group 2	Group 3
Nalbuphine consumption during first 24 h after surgery, mg	0 [0–5]	15 [10–20]^3^	20 [15–25]^3^
Total nalbuphine consumption during hospitalization, mg	5 [0–10]	45 [40–50]	50 [40–60]
Pain at rest in the first 24 h after surgery, NPRS	3 [2–4]	5 [3–6]	6 [4–7]
Pain during movements in the first 24 h after surgery, NPRS	4 [3–5]	6 [4–7]	7 [5–8]
Number of patients with severe pain, n (%)	3/27 (10)^1^	14/16 (47)	18/12 (60)^1^
Analgesia on-demand, n	0 [0–1]^1^	3 [2–4]	4 [3–4]
Sleep quality (0 to 10)	8 [7–9]^1^	6 [5–7]	4 [3–5]
Mobilization (sitting) on 1st day, n (%)	20/10 (67)^2^	10/20 (33)	3/27 (10)
Mobilization (getting up) on 1st day, n (%)	4/26 (13)	0/30	0/30
Mobilization (sitting) on 2nd days, n (%)	29/1 (3)	25/5 (83)	20/10 (67)^1^
Mobilization (getting up) on 2nd days, n (%)	27/3 (90)^2^	10/20 (33)	8/22 (27)

Unless specified otherwise, values are expressed as medians, with 25–75% interquartile ranges. *NRS* numeric pain rating scale. ^*1*^ *–* Fisher’s exact test, *p < 0,05;* ^*2*^ *–* Fisher’s exact test, *p < 0,001,* ^*3*^*-* Mann-Whitney test

No serious complications or side effects were reported. The study groups had no significant differences in the incidence of hypertension, bradycardia, tachycardia (Table 3). Hypotension was significantly more common in group 2 spinal anaesthesia (OR 9 95 CI 1.9–47, *p* = 0.004). Nausea and vomiting occurred significantly more often in control group 3 (general anaesthesia) compared with the study group 1 (OR 7 95 CI 1,3–35, *p* = 0,02). MINS was diagnosed significantly more often in control groups 2 and 3 compared with study group 1 (OR 9 95 CI 1,01–77, p = 0,048 for group 2 and OR 11 95 CI 1,2–91, *p* = 0, 03 for group 3). However, none of the patients had symptoms of myocardial ischemia and / or myocardial infarction. Nosocomial pneumonia was diagnosed in 1 patient in group 2, and 4 patients in group 3, no significant difference in the risk of nosocomial pneumonia was found. Detailed information on the frequency of complications in the groups is given in the Table 3.

**Table 3:** Frequency of complications and side effects in groups

Indicator / group	Group 1	Group 2	Group 3	*p*
Hypotension, n (%)	1/29	12/18^1^	4/26	–
Hypertension, n (%)	2/28	1/29	2/28	*p* > 0,05
Bradycardia, n (%)	1/29	3/27	1/29	*p* > 0,05
Tachycardia, n (%)	4/26	2/28	1/29	*p* > 0,05
MINS, n (%)	1/29^1^	7/23	8/22	–
Nosocomial pneumonia, n (%)	0/30	1/29	4/26	*p* > 0,05
Delirium, n (%)	0/30	1/29	1/29	*p* > 0,05
Nausea / vomiting, n (%)	2/28	5/25	10/20^1^	–
Itching, n (%)	0/30	1/29	2/28	*p* > 0,05

Fisher’s exact test*: 1 – p < 0,05; 2 – p < 0,001*

## Discussion

Perioperative analgesia in elderly patients with fractures of the proximal femur become a challenge due to risks of cardiac, thrombotic, pulmonary complications, high comorbidity incidence and severity, already prescribed drug therapy (anticoagulants). Although effective pain management in this patients group plays a key role in early mobilization, decreasing the complications rate, including delirium, survival and life expectancy after injury.

In our randomized controlled trial, we compared the perioperative use of the psoas compartment block with other techniques of anesthesia - spinal and general anesthesia with systemic analgesia before and after surgery. According to the results of this study, the most effective analgesic technique was prolonged compartment psoas block, started from admission to the hospital and continued postoperatively. PCB was associated with significantly lower number of patients with severe pain, lower opioid consumption and lower on demand analgesia incidence, better sleep quality, and faster mobilization, lower risk of opioid-associated side effects (nausea and vomiting) and MINS. We did not find a difference in the nosocomial pneumonia and delirium incidence.

Another studies had similar results, Canakci et al. [14] reported, that the psoas compartment block (PCB) provide longer time to first analgesia, comparing with the spinal anesthesia (SA). Although PCB group had significantly lower opioid consumption – 300 mg tramadol versus 1500 mg in SA group. In our study PCB patients also had significantly lower opioid (nalbufin) consumption, comparing with both control groups (Table 2).

Meta-analysis of 31 trials published in 2017 [15] showed that peripheral nerve blocks reduced pain on movement within 30 min of block placement, in this study we also showed the efficacy of PCB for pain management after femur surgery – the number of patients with severe pain and analgesia on demand was significantly lower in PCB group. They also did not find a difference in the risk of acute confusional state (risk ratio (RR) 0.69, 95% CI 0.38 to 1.27; I^2^ = 48%), as we didn’t too in this study. Three trials with 131 participants reported decreased risk for pneumonia (RR 0.41, 95% CI 0.19 to 0.89; I^2^ = 3%), in our study we didn’t find this, maybe due to not large enough study groups. The authors did not find a difference in risk of myocardial ischaemia or death within 6 months, but the number of participants included was well below the optimal information size for these two outcomes. In our study we also did not find this difference, although the incidence of MINS was significantly lower in PCB group versus SA and GA groups. Two trials with 155 participants reported that peripheral nerve blocks also reduced time to first mobilization after surgery (mean difference − 11.25 h, 95% CI − 14.34 to - 8.15 h; I^2^ = 52%), the same results we had in our study – number of patients, who was mobilizated in the 1st day (sitting) and 2nd day (getting up) after surgery was significantly higher in the PCB group.

The limitations of this study include the partially blinded design with absence of placebo control and the small sample size (*n* = 90), which make it difficult to made final conclusions about efficacy and safety of the PCB.

Nevertheless, this trial showed that perioperative PCB for elderly patients with proximal femur fracture, is effective to decrease the number of patients with severe pain, the number of on demand analgesia and opioid consumption. PCB also reduce the incidence of opioid-associated nausea and vomiting, the incidence of MINS and increase number of patients, who could sit in the 1st day after surgery and get up on the 2nd day.

## Conclusion

Perioperative PCB in elderly patients with a proximal femur fracture could be an effective analgesia technique, as it decrease the number of patients with severe pain, need for on demand analgesia and opioid consumption. PCB also decrease the incidence of opioid-associated nausea and vomiting, comparing to general anesthesia, and increase the number of patients, who was mobilized in the 1st day (sitting) and 2nd day (getting up) after surgery. PCB may reduce the incidence of MINS, although to assess this outcome more studies are needed.

## Supplementary Information

**Additional file 1.**

## Data Availability

Reasonable requests for access to the datasets used and/or analysed during the study can be made to the corresponding author.

## Abbreviations

ASA: American Society of Anesthesiology
BIS: Bispectral index
COPD: Chronic obstructive pulmonary disease
ECG: Electrocardiogram
FNB: Femoral nerve block
GA: General anesthesia
ICU: Intensive care unit
NRPS: Numeric rating pain scale
NSAIDs: Nonsteroidal anti-inflammatory drugs
PCB: Psoas compartment block
RASS: Richmond Agitation-Sedation Scale
SA: Spinal anesthesia
USA: Underwent spinal anesthesia
VRS: Verbal rating scale
