# Supplementary material for: Psoas compartment block efficacy and safety for perioperative analgesia in the elderly with proximal femur fractures: a randomized controlled study
Source: BMC Anesthesiol. 2021 Oct 25;21:252. doi: 10.1186/s12871-021-01473-9 (PMC8546936; doi:10.1186/s12871-021-01473-9)
Supplement: Supplementary file 1 — Additional file 1. [file 12871_2021_1473_MOESM1_ESM.docx]

**Meeting inclusion criteria, provided consent** n=95

**Excluded n=5**

chronic heart failure NYHA III - 2

respiratory failure - 2

liver failure C.-P. class C – 1

**Randomized** n=90 Patients who planned osteosynthesis of the proximal femur and who met the inclusion criteria were randomized to 3 study groups in a 1:1 ratio (Figure 1) using random assignment in blocks of four. The randomization sequence was generated using a computer algorithm [19]. Randomization and data analysis were conducted by an independent blinded member of the research team.


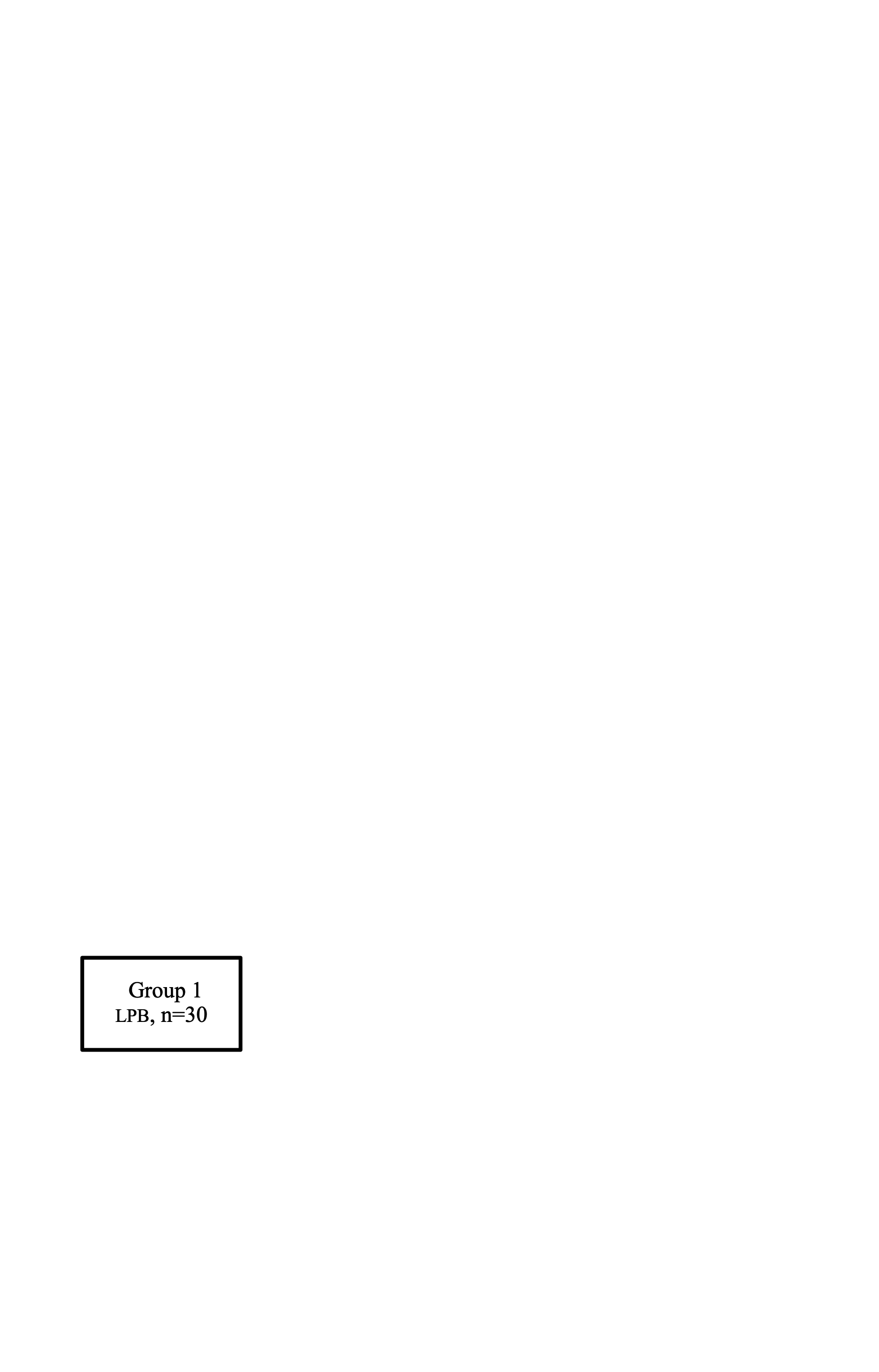

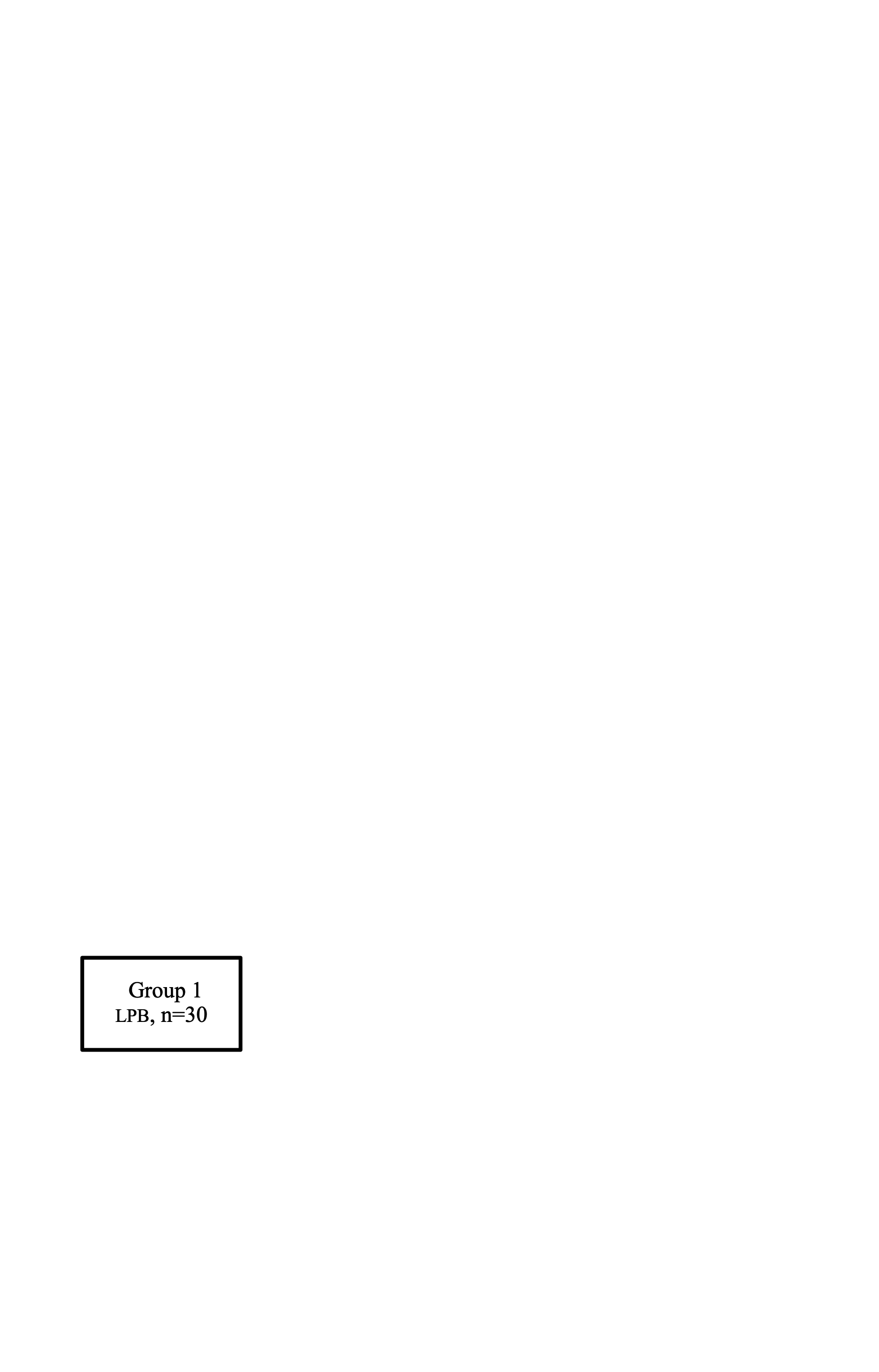
 patients

Group 3 (GA) n=30

Group 2 (SA) n=30

Group 1 (PCB)

**Analyzed** n=30

**Analyzed** n=32

**Analyzed** n=30
